# Visual Function Assessment in Simulated Real-Life Situations in HIV-Infected Subjects

**DOI:** 10.1371/journal.pone.0097023

**Published:** 2014-05-08

**Authors:** Giulio Barteselli, Jay Chhablani, Maria Laura Gomez, Aubrey L. Doede, Laurie Dustin, Igor Kozak, Dirk-Uwe Bartsch, Stanley P. Azen, Scott L. Letendre, William R. Freeman

**Affiliations:** 1 Department of Ophthalmology, Jacobs Retina Center at Shiley Eye Center, University of California San Diego, La Jolla, California, United States of America; 2 Ophthalmological Unit, Department of Clinical Sciences and Community Health, Ca’ Granda Foundation-Ospedale Maggiore Policlinico, University of Milan, Milan, Italy; 3 Srimati Kanuri Santhamma Vitreo-Retina Service, L.V. Prasad Eye Institute, Hyderabad, India; 4 Department of Preventive Medicine, Keck School of Medicine, University of Southern California, Los Angeles, California, United States of America; 5 King Khaled Eye Specialist Hospital, Riyadh, Kingdom of Saudi Arabia; 6 HIV Neurobehavioral Research Center, University of California San Diego, La Jolla, California, United States of America; Eye Hospital, Charité, Germany

## Abstract

Visual function abnormalities are common in people living with HIV disease (PLWH) without retinitis, even after improvement in immune status. Abnormalities such as reduced contrast sensitivity, altered color vision, peripheral visual field loss, and electrophysiological changes are related to a combination of retinal dysfunctions, involving inner and outer retinal structures. The standard protocol for testing vision performance in clinical practice is the Early Treatment Diabetic Retinopathy Study (ETDRS) chart. However, this method poorly correlates with activities of daily living that require patients to assess visual stimuli in multiple light/contrast conditions, and with limited time. We utilized a novel interactive computer program (Central Vision Analyzer) to analyze vision performance in PLWH under a variety of light/contrast conditions that simulate stressful and real-world environments. The program tests vision in a time-dependent way that we believe better correlates with daily living activities than the non-timed ETDRS chart. We also aimed to correlate visual scores with retinal neuro-fiber layer thickness on optical coherence tomography. Here we show that visual acuity is more affected in PLWH in comparison to HIV-seronegative controls in varying contrast and luminance, especially if the nadir CD4+ T-cell count was lower than 100 cells/mm^3^. Visual impairment reflects the loss of retinal nerve fiber layer thickness especially of the temporal-inferior sector. In PLWH the ETDRS chart test led to better visual acuity compared to the Central Vision Analyzer equivalent test, likely because patients had indefinite time to guess the letters. This study confirms and strengthens the finding that visual function is affected in PLWH even in absence of retinitis, since we found that the HIV serostatus is the best predictor of visual loss. The Central Vision Analyzer may be useful in the diagnosis of subclinical HIV-associated visual loss in multiple light/contrast conditions, and may offer better understanding of this entity called “neuroretinal disorder”.

## Introduction

Visual function abnormalities are common in people living with human immunodeficiency virus (HIV) disease (PLWH) without retinitis, even after improvement in immune status with antiretroviral therapy (ART). [1], [2] Abnormalities include reduced contrast sensitivity, altered color vision, peripheral visual field loss and electrophysiological changes.[3]–[9] These changes in visual function are thought to be caused by HIV-associated “neuroretinal disorder,” which is characterized by damage in the retinal nerve fiber layer (RNFL), as detected by optical coherence tomography (OCT), most likely due to microinfarctions and microangiopathy. [10] Although the exact pathogenesis of HIV-related microangiopathy remains uncertain, several hypotheses have been reported including direct HIV infection of vascular endothelial cells, damage from immune complexes, and rheological abnormalities. [11] Moreover, recent data from our group showed dysfunction of the outer retinal layers, especially photoreceptors and retinal pigment epithelium, in autopsy eyes of HIV-seropositive donors. [12] Therefore, there may be a second separate mechanism of vision dysfunction in these eyes.

Since their introduction to clinical practice, the Snellen chart test and the more recent Early Treatment Diabetic Retinopathy Study (ETDRS) chart test have been the standard protocol for testing best-corrected visual acuity (BCVA). [13] However, these tests are unable to detect subtle visual loss, especially under low contrast or glare conditions. Indeed, it is generally recognized that the ETDRS chart test poorly correlates with activities of daily living [14] (such as driving at night or playing sports outside), since it’s not time-dependent, and as it allows an evaluation of the BCVA only in a pre-determined single high-contrast glare environment. [15] If the widely used ETDRS chart test is poorly reliable in evaluating BCVA in other contrast or luminance conditions for normal eyes, this may be also worse for PLWH. Indeed, these patients have a poor low-contrast visual performance – even without any retinitis [2] – and can also have poor driving performance. [16], [17] Thus, a method able to measure the visual function in varying contrast and glare conditions and in time-dependent situations is needed.

The Central Vision Analyzer (CVA; Sinclair Technologies, LLC, Media, PA) is a new interactive computer program to analyze BCVA under conditions that simulate stressful and real-world environments. [18], [19] This backlight-glare computer-based test for BCVA is able to simulate luminance, contrast, and glare conditions that one may experience in a variety of daily activities, such as glare, dim lighting, and night vision. Moreover, the CVA testing is a time-dependent technique, yielding a better assessment of real-life encounters. Therefore, it may be an ideal tool to assess visual function and to understand the visual behavior under real-life situations. The usefulness of this device has been tested in a registered study at http://clinicaltrials.gov (identification, NCT 02028351), but results are not yet published.

The present study of PLWH aims to 1) assess visual acuity measurements in simulated real-life situations using the CVA; 2) correlate visual scores with RNFL thickness on spectral-domain OCT; and 3) assess the best predictors of visual performance under different light/contrast conditions using the CVA.

## Methods

### Subjects

This prospective, cross-sectional study included 89 eyes of 47 PLWH without active or healed retinitis and 105 eyes of 57 HIV-seronegative adults (Table 1). The PLWH were part of a cohort consecutively recruited from the University of California San Diego (UCSD) AIDS Ocular Research Unit at the Jacobs Retina Center in La Jolla, CA from June 2010 to June 2011. The PLWH were recruited during the annual visit scheduled for research purpose; none of the patients had any ocular symptoms at enrollment. The HIV-seronegative individuals were recruited from the local community. This study was approved by the UCSD Human Research Protections Program and followed the tenets of the Declaration of Helsinki. Signed informed consent was obtained from all subjects before enrollment.

**Table 1:** Characteristics of each of the CVA modules tested.

CVA modules	Contrast (MC)	Landolt C Luminance	Background luminance	Simulated Environment
*Mesopic 1 (M1)*	99%	220 Cd/m^2^	3 Cd/m^2^	Full contrast
*Mesopic 2 (M2)*	64%	4.8 Cd/m^2^	3 Cd/m^2^	Dim restaurant
*Mesopic 3 (M3)*	43%	8.4 Cd/m^2^	3 Cd/m^2^	Driving at dusk
*Glare 1 (G1)*	99%	1.6 Cd/m^2^	200 Cd/m^2^	Full contrast
*Glare 2 (G2)*	10%	180 Cd/m^2^	200 Cd/m^2^	Outside with sun over head
*Glare 3 (G3)*	8%	186 Cd/m^2^	200 Cd/m^2^	Outside with sun 15° off-axis

CVA, Central Vision Analyzer; MC, Michelson Contrast; Cd/m^2^, Candelas per Meter Squared.

PLWH were divided into two groups based on the nadir CD4+ T-cell count. The high-nadir CD4 group maintained CD4+ T-cell counts above 200 cells/mm^3^. From this group, 37 eyes from 19 persons were studied; 1 eye was excluded because of lens opacities. The low-nadir CD4 group included PLWH with nadir CD4+ T-cell counts lower than 200 cells/mm^3^ for at least 6 months in their medical history. In this group, 52 eyes from 28 patients were studied; 4 eyes were excluded because of a history of infectious retinitis. All PLWH were taking ART for at least 6 months prior to the time of the examination. The exclusion criteria included history of ocular opportunistic infections; visible ocular abnormalities on indirect ophthalmoscopy or slit-lamp ophthalmoscopy; intraocular pressure 22 mmHg or higher; spherical equivalent refractive error below −5 diopters or above +2.5 diopters; and concurrent disease that could cause retinal damage, such as diabetes or glaucoma. In the HIV-seronegative control group, 105 eyes from 57 volunteers were studied; 3 eyes were excluded because of significant lens opacities; 6 eyes were excluded because of macular pathology affecting vision.

### Ocular Examination and Central Vision Analyzer Testing

All patients had a complete ocular examination, including BCVA examination using standard ETDRS charts, slit-lamp examination, intraocular pressure measurement, indirect ophthalmoscopy under dilated pupils, and peripapillary RNFL thickness measurement on Heidelberg Spectralis (Heidelberg Engineering, Carlsbad, CA).

Before dilation, patients underwent CVA testing using a Landolt C presentation of 900 msec in 6 different light/contrast conditions (Table 1). The Landolt C appeared in three mesopic conditions of 99% contrast against 3 Cd/m2 background (*“M1” module*, that is full-contrast module of white letters presented on a black background), followed by lower contrast modules of 64% contrast (*“M2” module*, simulating an environment similar to a dimly lit restaurant) and 43% contrast (*“M3” module*, simulating an environment similar to driving at dusk). Photopic glare (backlighting) conditions were simulated using 99% contrast against a 200 Cd/m2 background (*“G1” module*, that is a full-contrast module of black letters presented on a bright background), 10% contrast (*“G2” module*, simulating an environment similar to playing a sport outside with the sun over head), and 8% contrast (*“G3” module*, simulating an environment similar to playing a sport outside with the sun 15 degrees off-axis). The CVA displayed a tumbled Landolt C in 4 different directions on a monitor positioned 4 meters from the patient, and to which the person responds by pressing one of 4 buttons on a keypad (corresponding to the 4 different orientations). The program utilized a 0.05 logMAR staircase of optotype size and thresholds for the smallest Landolt C for which the person accurately identifies the tumbled position twice with 2 inaccurate responses at the next smaller size. At the end of the test, results were automatically presented to the examiner in a report as six separate BCVA scores in logMAR units for each of the simulated light/contrast conditions. [10].

### Statistical Analysis

Mean age and duration of HIV were compared between HIV groups using independent samples t-tests and chi-square tests were used to test for gender differences between groups. Generalized Estimating Equations (GEE) were used to compare LogMAR BCVA and RNFL measures between HIV groups, adjusting for gender. Bonferonni adjustments were made for multiple pairwise comparisons. GEE was also used for the gender-adjusted univariate and multivariate analyses, testing for the independent association of HIV status and RNFL with logMAR BCVA. Statistical analyses were performed using SAS statistical software version 9.3 (SAS Inc, Cary, North Carolina, USA). A p-value <0.05 was considered to be statistically significant.

## Results

### Demographic and Disease Characteristics

At the time of examination, all 47 PLWH showed near-normal immune status with ART. The mean CD4+ T-cell count was 672±281 cells/mm^3^ (range, 264–1,305 cells/mm^3^) and the mean HIV plasma viral load was 24.9±19.9 copies/mL (range, 0–50 copies/mL). Table 2 presents groups demographics. The two HIV groups (i.e., low-nadir CD4 group and high-nadir CD4 group) were similar for age (p = 0.66), as were the HIV-seropositive and HIV-seronegative groups (p = 0.45). The HIV-seropositive group included more men than women compared to the HIV-seronegative group (p = 0.03). The duration of HIV disease was similar between the two HIV groups (p = 0.62).

**Table 2:** Demographics of Study Participants.

Variable	Low CD4*	High CD4	p-value	HIV+	HIV−	p-value	Total
No. of subjects	28	19		47	57		104
No. of eyes	52	37		89	105		194
Mean age (y)	55.1	53.9	0.66^T^	54.6	56.2	0.45^T^	55.5
Age range (y)	41–85	44–73		41–85	28–84		28–85
Men (%)	79%	100%	0.03^C^	87%	68%	0.03^C^	77%
Women (%)	21%	0%		13%	32%		23%
Duration of HIV (y)	18.2	17.0	0.62^T^	17.7	n/a		n/a

Y, years; n/a, not applicable;

*CD4+ T-cell counts lower than 200 cells/mm3 for at least 6 months in their medical history;

T = Independent samples t-test;

C = Chi-square test.

### Assessment of Visual Acuity Measurements using the Central Vision Analyzer

Comparing visual acuity measurements for various mesopic and backlight-glare conditions among the 3 groups (Table 3), we found statistically significant differences for ETDRS (p = 0.029), M1 (p<0.001), M2 (p = 0.021) and M3 (p = 0.029), and G1 modules (p = 0.001), after adjusting for gender. In particular, the high-nadir CD4 group experienced worse visual scores than HIV-seronegative subjects for ETDRS (p = 0.028) and G1 scores (p<0.001). The low-nadir CD4 group experienced worse visual scores than HIV-seronegative subjects for ETDRS (p = 0.058), M1 (p = 0.001), M2 (p = 0.012) and M3 (p = 0.021), and G1 modules (p<0.001). The acuity drop between ETDRS and G1 was also significantly different between groups (p = 0.005); in particular, it was greater in the low-nadir CD4 group than in HIV-seronegative subjects (p = 0.001). Among PLWH, the two groups had similar BCVA scores (p>0.05). Gender-adjusted Spearman correlation coefficients with visual scores among PLWH showed that G1 scores were correlated with vision decrease (p<0.001), while EDTRS scores were not (p = 0.100). No significant correlation was found between presumed duration of HIV and visual scores in any CVA module.

**Table 3:** Analysis of visual acuity scores between HIV-seronegative and HIV-seropositive patients, adjusted for gender.

		HIV− (57)	HIV+ (47)			HIV− vs high CD4	HIV− vs low CD4	High vs low CD4
BCVA (logMAR)			low CD4 (28)	high CD4 (19)	p-value*	p-value*	p-value*	p-value*
*ETDRS*	*Lsmean*	−0.067	−0.008	−0.015	**0.029**	**0.028**	0.058	0.843
	*SE*	0.013	0.029	0.019				
*M1*	*Lsmean*	−0.043	0.140	0.129	**<0.001**	**<0.001**	**0.001**	0.863
	*SE*	0.02	0.045	0.046				
*M2*	*Lsmean*	0.194	0.334	0.309	**0.021**	0.078	**0.012**	0.736
	*SE*	0.030	0.048	0.057				
*M3*	*Lsmean*	0.313	0.462	0.434	**0.029**	0.070	**0.021**	0.726
	*SE*	0.033	0.057	0.057				
*G1*	*Lsmean*	0.018	0.191	0.102	**0.001**	0.161	**<0.001**	0.195
	*SE*	0.026	0.042	0.053				
*G2*	*Lsmean*	0.354	0.429	0.399	0.421	0.444	0.240	0.699
	*SE*	0.028	0.059	0.049				
*G3*	*Lsmean*	0.446	0.546	0.456	0.362	0.885	0.158	0.291
	*SE*	0.035	0.063	0.055				
*ETDRS - G1*	*Lsmean*	−0.085	−0.199	−0.118	**0.005**	0.528	**0.001**	0.134
	*SE*	0.023	0.027	0.046				

*Applying Generalized Estimating Equations (GEE); BCVA, best corrected visual acuity; SE, standard error; ETDRS, Early Treatment Diabetic Retinopathy Study; M1, full-contrast module of white letters presented on a black background; M2, 64% contrast module simulating an environment similar to a dimly lit restaurant; M3, 43% contrast module simulating an environment similar to driving at dusk; G1, full-contrast module of black letters presented on a bright background; G2, 10% contrast module simulating an environment similar to playing a sport outside with the sun over head; G3, 8% contrast module simulating an environment similar to playing a sport outside with the sun 15 degrees off-axis.

In addition, we explored the hypothesis that the vision dysfunction has a common pathway to neurophysiological dysfunction. Changing the definition of low-nadir CD4 value from 200 to 100 cells/mm^3^, statistical analysis showed more robust results (data not shown); the high-nadir CD4 group (over 100 cells/mm^3^) experienced worse visual scores than HIV-seronegative subjects not only for ETDRS (p = 0.039) and G1 scores (p<0.003) as we described above, but also for M1 (p = 0.001), M2 (p = 0.014) and M3 (p = 0.020).

### Correlation of Visual Scores with RNFL Thickness on Spectral-domain OCT

After correcting for gender (Table 4), the global peripapillary RNFL thickness (“G” value on the Heidelberg Spectralis) was similar between groups (p = 0.353). The only RNFL sector that showed difference between groups was the nasal-superior (p = 0.039); the low-nadir CD4 group had thinner RNFL compared the high-nadir CD4 group (p = 0.021) as well as compared to HIV-seronegative subjects (p = 0.041). No correlation was noted between duration of HIV disease and RNFL thickness.

**Table 4:** Analysis of retinal nerve fiber layer thickness between HIV-seronegative and HIV-seropositive patients, adjusted for gender.

		HIV− (57)	HIV+ (47)			HIV− vs high CD4	HIV− vs low CD4	High vs low CD4
RNFL (µm)			low CD4 (28)	high CD4 (19)	p-value*	p-value*	p-value*	p-value*
*“G” (global)*	*Lsmean*	94.6	91.0	92.7	0.353	0.604	0.155	0.663
	*SE*	1.3	2.1	3.3				
*Temp*	*Lsmean*	69.7	65.7	64.2	0.187	0.095	0.230	0.707
	*SE*	1.6	2.9	2.8				
*Temp-Sup*	*Lsmean*	123.5	118.5	123.0	0.645	0.941	0.359	0.560
	*SE*	3.2	4.4	6.1				
*Nas-Sup*	*Lsmean*	105.7	95.8	109.2	**0.039**	0.530	**0.041**	**0.021**
	*SE*	3.2	3.7	4.3				
*Nas*	*Lsmean*	70.7	72.2	76.3	0.497	0.237	0.726	0.439
	*SE*	2.2	3.5	4.2				
*Nas-Inf*	*Lsmean*	113.7	107.4	98.0	0.071	**0.022**	0.337	0.207
	*SE*	3.7	5.2	5.5				
*Temp-Inf*	*Lsmean*	132.2	126.2	128.7	0.406	0.624	0.180	0.722
	*SE*	2.9	3.2	6.4				

*Applying Generalized Estimating Equations (GEE); RNFL, retinal nerve fiber layer thickness; SE, standard error.

Changing the definition of low-nadir CD4 value from 200 to 100 cells/mm^3^, statistical analysis showed more robust results (data not shown); the global peripapillary RNFL thickness turned out to be significantly different between groups (p = 0.032), and in particular it was thinner in the low-nadir CD4 group compared to HIV-seronegative subjects (p = 0.011).

### Assessment of the Best Predictors of Visual Performance

Gender-adjusted univariate regression analysis (Table 5) confirmed that the HIV serostatus was highly associated with visual scores for M1 (p<0.001), M2 (p = 0.006), M3 (p = 0.008), and G1 (p<0.001), as well as for ETDRS (p = 0.012). The global RNFL thickness was associated with visual scores for M1 (p = 0.040), M2 (p = 0.018), and M3 (p = 0.005). The temporal-inferior RNFL sector was the most associated to the vision performance: thinning of temporal-inferior RNFL sector was associated to visual scores for M1 (p = 0.033) and M2 modules (p = 0.016). A trend of association was found between thinning of temporal-inferior RNFL sector and visual scores for M3 (p = 0.064) and for ETDRS (p = 0.065).

**Table 5:** Regression analyses of HIV status (seronegative vs seropositive) and retinal nerve fiber layers thickness with visual acuity, adjusted for gender.

		Univariate		Multivariate	
BCVA (logMAR)	RNFL	HIV status	RNFL	HIV status	RNFL
		p-value*	p-value*	p-value*	p-value*
*M1*	*“G” (global)*	**<0.001**	**0.040**	**<0.001**	0.055
	*Temp-Inf*	**<0.001**	**0.033**	**<0.001**	**0.033**
*M2*	*“G” (global)*	**0.006**	**0.018**	**0.031**	**0.037**
	*Temp-Inf*	**0.006**	**0.016**	**0.023**	**0.032**
	*Temp-Sup*	**0.006**	0.123	**0.017**	0.130
*M3*	*“G” (global)*	**0.008**	**0.005**	**0.029**	**0.010**
	*Temp-Inf*	**0.008**	0.064	**0.019**	0.098
	*Nasal-Sup*	**0.008**	0.076	**0.021**	0.117
*G1*	*“G” (global)*	**<0.001**	**0.047**	**0.007**	0.058
	*Nasal-Sup*	**<0.001**	0.057	**0.006**	0.074
*ETDRS*	*“G” (global)*	**0.012**	**0.016**	**0.012**	0.268
	*Temp-Inf*	**0.012**	0.065	**0.010**	0.081

*Applying Generalized Estimating Equations (GEE); BCVA, best corrected visual acuity; RNFL, retinal nerve fiber layer; ETDRS, Early Treatment Diabetic Retinopathy Study; M1, full-contrast module of white letters presented on a black background; M2, 64% contrast module simulating an environment similar to a dimly lit restaurant; M3, 43% contrast module simulating an environment similar to driving at dusk; G1, full-contrast module of black letters presented on a bright background.

Gender-adjusted multivariate regression analysis (Table 5) showed that HIV serostatus was the best predictor of visual performance under different light/contrast conditions and correlated well with ETDRS (p = 0.012), M1 (p<0.001), M2 (p = 0.031), M3 (p = 0.029), and G1 modules (p = 0.007). Thickness of the temporal-inferior RNFL sector was also an independent predictor of visual performance, especially for M1 (p = 0.033) and M2 modules (p = 0.032).

## Discussion

This study documented visual function abnormalities and RNFL damage in PLWH without retinitis and confirmed findings of other studies. PLWH have been reported to present reduced contrast sensitivity, altered color vision, peripheral visual field loss and electrophysiological changes [3]–[9]. In our study, the visual function of HIV-seropositive and HIV-seronegative patients was comprehensively analyzed in several contrast and luminance conditions using a unique device – the Central Vision Analyzer – that has been already approved by the Food And Drug Administration. This new interactive computer program measures logMAR visual acuity under conditions that simulate stressful and real-world environments [18].

We demonstrated that visual scores are worse in PLWH in comparison to HIV-seronegative subjects in most of the CVA light/contrast conditions, especially if patients had history of nadir CD4+ T-cell count lower than 100 cells/mm^3^. The HIV serostatus turned out to be the best independent predictor of visual performance under different light/contrast conditions. We have already demonstrated that visual scores decline with decreasing contrast and luminance conditions both in healthy eyes and in eyes with macular pathology (Gomez ML, et al. IOVS 2011,52;ARVO E-Abstract 5555). Moreover, in that study we found that the drop in visual scores with decreasing light and contrast does not differ between these two groups, even if the visual outcome is worse for patients with macular pathology. In eyes with age-related macular degeneration, damage of the outer retinal layers is the principal cause of low visual outcomes. [20] In eyes of PLWH, visual disturbances are mainly related to damage of the inner retinal layers such as the RNFL, as detected by OCT, most likely due to microinfarctions and microangiopathy. [10] In addition, recent evidence has demonstrated a significant dysfunction of the outer retinal layers in autopsy eyes of HIV-seropositive donors even in the absence of infection or clinically apparent retinal lesions. [12] Therefore we can conclude that the lower visual outcomes in eyes of PLWH compared to HIV-seronegative controls may be related to a combination of retinal dysfunctions, involving both inner and outer retinal structures.

The luminance and contrast of the G1 module is the most similar to that of the ETDRS chart test; however in PLWH we found that the ETDRS chart test led to better visual acuity compared to the G1 test. This finding is likely due to the time-dependent nature of the automated CVA testing. Since the EDTRS chart is not time-dependent, patients have indefinite time to visualize letters on the chart; however this approach does not mimic real-world situations. The CVA testing simulates stressful and real-world environments, recreating light and contrast conditions of time-dependent real life activities such as reading, driving, or recognizing objects that are presented transiently. Therefore, if letters are projected only for a short period of time, patients with macular pathology may fail to recognize them since they have slower reading speed than healthy patients. [21] Similarly, PLWH may not be able to speedily recognize letters because of HIV-related cognitive impairment, which is common and can affect psychomotor speed and executive function. [22].

Although the nadir CD4+ T-cell count reflects the past severity of the immune disease, in our study we did not find significantly reduced visual scores in the low-nadir CD4 group compared to the high-nadir CD4 group in any of the several light and contrast conditions. Interestingly, the visual drop in high-contrast environments (i.e. ETDRS and G1) was also similar between HIV-seropositive groups. Previous reports have described abnormalities on visual field and multifocal electroretinogram (mfERG) in eyes of PLWH in the absence of infectious retinitis; patients with reduced immune status differed more from healthy eyes than eyes from patients who never were observed to have reduced immune status. [8], [23] However, a subsequent study using more powerful data analysis showed equal severity of b-latency abnormalities on mfERG in the low- and high-CD4 groups. [24] The similarity of mfERG abnormalities in the low- and high-nadir CD4 groups [24], as well as the similar visual loss detected by the CVA as described in the present study, indicates that good immune status during ART may not protect against retinal damage. However, we also can hypothesize that the immune status as indicated by the nadir CD4+ T-cell count may not be the best predictor of visual dysfunctions in HIV-positive subjects. Many other factors may be implicated, such as current and highest HIV RNA level, duration of HIV RNA suppression over time, number of opportunistic infections, neurocognitive function, and also distribution of ART drugs into the central nervous system. [25] Although we know the date of diagnosis of HIV seropositivity, some patients on presentation already had HIV for many years. This may explain why we did not find correlations between duration of HIV seropositivity and visual scores or RNFL thickness. A more important predictor of visual performance and retinal damage may be knowledge of the duration of low CD4+ T-cell counts and uncontrolled HIV RNA. Such data is extremely difficult to procure. Although we have some of these data on a small number of our patients at the moment, we do not have sufficient data for our analysis. Therefore, in this pilot analysis, we included all patients and classified them based on the limited data currently available.

We also found thinning of the global peripapillary RNFL in the low-nadir CD4 group compared to HIV-seronegative subjects, as well as association between global RNFL and visual scores for most CVA modules. Regression analysis indicated that the temporal-inferior RNFL sector was the most associated sector with visual performance in PLWH. This is consistent with previous studies: a strong relationship was found between contrast sensitivity and color vision with peripapillary RNFL in PLWH, most apparent in the temporal quadrant. [26] These results are biologically plausible; BCVA, contrast sensitivity, and color vision are macular functions, and it is not surprising that correlations were found primarily in the temporal RNFL, which reflects the papillo-macular bundle. Because clinically we see a similar distribution of cotton-wool spots in HIV-seropositive patients with low-CD4 counts, we assume that these retinal microinfarctions are at least one factor responsible for subsequent defects in RNFL thickness. Indeed, histopathologic and tomographic studies showed significance of cotton-wool spots in pathogenesis of retinal neural tissue loss. [27], [28].

There are a number of limitations to this study. Sample sizes were relatively small, which may have affected our ability to identify some potential associations. Certain components of study participants’ medical history was self-reported and approximate, such as date of HIV seroconversion, total number of opportunistic infections, and ART duration and type over time. A comprehensive analysis of ART is not possible in our population and might prove extremely difficult because ART protocols are changed frequently; longitudinal studies are necessary to assess the effect of ART drugs on visual function. In addition, knowing the changes in HIV RNA level overtime would be useful for further analysis. Unfortunately, because of the long duration of HIV infection in our study population (average duration: 17 years) and because of the relatively recent referral to our center, we do not have precise data on fluctuations of the HIV RNA level in our population. This should be a topic of future studies. However, we found strong and consistent results and we believe that it is unlikely that they would be observed based on study deficiencies as opposed to real effects.

In conclusion, in our study we analyzed the visual function in PLWH without active retinitis using a novel interactive computer program that is able to test visual acuity in a variety of real-life mesopic and glare conditions. We demonstrated that the HIV status (positive vs negative) is the best independent predictor of visual performance under different light/contrast conditions. Visual function is more affected in PLWH in comparison to HIV-seronegative subjects in varying contrast and luminance, especially if patients had history of nadir CD4+ T-cell count lower than 100 cells/mm^3^, and reflects the loss of RNFL thickness especially of the temporal-inferior sector. This study confirms, strengthens, and implements the previous finding that visual function is affected in PLWH even in absence of retinitis. We suggest paying particular attention while evaluating PLWH, even if the immune system is near-normal at the time of examination, because they may experience a greater visual dysfunction than expected. The CVA, or a similar time-dependent vision testing, may be of clinical use in the non-invasive diagnosis of early subclinical HIV-associated visual dysfunction, and may offer better understanding of this entity called “neuroretinal disorder”.

## References

[1] Freeman WR, Van Natta ML, Jabs D, Sample PA, Sadun AA, et al (2008) Vision function in HIV-infected individuals without retinitis: report of the Studies of Ocular Complications of AIDS Research Group. Am J Ophthalmol 145: 453–462. 18191094 10.1016/j.ajo.2007.10.013 PMC2359724

[2] Mueller AJ, Plummer DJ, Dua R, Taskintuna I, Sample PA, et al (1997) Analysis of visual dysfunctions in HIV-positive patients without retinitis. Am J Ophthalmol 124: 158–67. 9262539 10.1016/s0002-9394(14)70780-9

[3] Mutlukan E, Dhillon B, Aspinall P, Cullen JF (1992) Low contrast visual acuity changes in human immuno-deficiency virus (HIV) infection. Eye (Lond) 6: 39–42. 1426397 10.1038/eye.1992.6

[4] Quiceno JI, Capparelli E, Sadun AA, Munguia D, Grant I, et al (1992) Visual dysfunction without retinitis in patients with acquired immunodeficiency syndrome. Am J Ophthalmol 113: 8–13. 1728151 10.1016/s0002-9394(14)75745-9

[5] Shah KH, Holland GN, Yu F, Van Natta M, Nusinowitz S, Studies of Ocular Complications of ARG (2006) Contrast sensitivity and color vision in HIV-infected individuals without infectious retinopathy. Am J Ophthalmol 142: 284–292. 16876510 10.1016/j.ajo.2006.03.046

[6] Sommerhalder J, Baglivo E, Barbey C, Hirschel B, Roth A, et al (1998) Colour vision in AIDS patients without HIV retinopathy. Vision Res 38: 3441–3446. 9893862 10.1016/s0042-6989(98)00011-x

[7] Falkenstein I, Kozak I, Kayikcioglu O, Cheng L, Bartsch DU, et al (2006) Assessment of retinal function in patients with HIV without infectious retinitis by multifocal electroretinogram and automated perimetry. Retina 26: 928–934. 17031295 10.1097/01.iae.0000250009.60908.35

[8] Kozak I, Sample PA, Hao J, Freeman WR, Weinreb RN, et al (2007) Machine learning classifiers detect subtle field defects in eyes of HIV individuals. Trans Am Ophthalmol Soc 105: 111–118. 18427600 PMC2258123

[9] Sample PA, Plummer DJ, Mueller AJ, Matsubara KI, Sadun A, et al (1999) Pattern of early visual field loss in HIV-infected patients. Arch Ophthalmol 117: 755–760. 10369585 10.1001/archopht.117.6.755

[10] Kozak I, Bartsch DU, Cheng L, Kosobucki BR, Freeman WR (2005) Objective analysis of retinal damage in HIV-positive patients in the HAART era using OCT. Am J Ophthalmol 139: 295–301. 15733991 10.1016/j.ajo.2004.09.039 PMC3757251

[11] Geier SA, Hammel G, Bogner JR, Kronawitter U, Berninger T, et al (1994) HIV-related ocular microangiopathic syndrome and color contrast sensitivity. Invest Ophthalmol Vis Sci 35: 3011–3021. 8206718

[12] Kozak I, Sasik R, Freeman WR, Sprague LJ, Gomez ML, et al (2013) A Degenerative Retinal Process in HIV-Associated Non-Infectious Retinopathy. PloS one 8: e74712. 24069333 10.1371/journal.pone.0074712 PMC3775801

[13] Lim LA, Frost NA, Powell RJ, Hewson P (2010) Comparison of the ETDRS logMAR, ‘compact reduced logMar’ and Snellen charts in routine clinical practice. Eye (Lond) 24: 673–677. 19557025 10.1038/eye.2009.147

[14] Pesudovs K, Hazel CA, Doran RM, Elliott DB (2004) The usefulness of Vistech and FACT contrast sensitivity charts for cataract and refractive surgery outcomes research. Br J Ophthalmol 88: 11–16. 14693761 10.1136/bjo.88.1.11 PMC1771933

[15] Yavuz GA, Unver YB, Bekiroglu N, Presti P, Sinclair SH (2009) Central field perimetry of discriminated targets: I. Results for normal individuals using high-contrast targets. Eye (Lond) 23: 2082–2089. 19648898 10.1038/eye.2009.177

[16] Cheng S KH, Bartsch DU, Kozak I, Marcotte TD, Freeman WR (2011) Relationship between retinal nerve fiber layer thickness and driving ability in patients with human immunodeficiency virus infection. Graefes Arch Clin Exp Ophthalmol 249: 1643–1647. 21732109 10.1007/s00417-011-1735-4 PMC4144406

[17] Holland GN, Kappel PJ, Van Natta ML, Palella FJ, Lyon AT, et al (2010) Association between abnormal contrast sensitivity and mortality among people with acquired immunodeficiency syndrome. Am J Ophthalmol 149: 807–816. 20399927 10.1016/j.ajo.2009.12.019 PMC4121666

[18] Gutstein W, Sinclair SH, North RV, Bekiroglu N (2010) Screening athletes with Down syndrome for ocular disease. Optometry 81: 94–99. 20152783 10.1016/j.optm.2009.09.017

[19] Gomez ML (2014) Measuring the Quality of vision after cataract surgery. In “Cataract surgery and lens implantation”. Curr Opin Ophthalmol 25: 3–11. 24225444 10.1097/ICU.0000000000000011

[20] El-Emam S, Chhablani J, Barteselli G, Wang H, Lee SN, et al (2013) Correlation of Spectral Domain Optical Coherence Tomography Characteristics with Visual Acuity in Eyes with Subfoveal Scarring after Treatment for Wet Age-Related Macular Degeneration. Retina 33: 1249–1257. 23446655 10.1097/IAE.0b013e31827b6439

[21] Nguyen NX, Weismann M, Trauzettel-Klosinski S (2009) Improvement of reading speed after providing of low vision aids in patients with age-related macular degeneration. Acta Ophthalmol 87: 849–853. 19141148 10.1111/j.1755-3768.2008.01423.x

[22] Manly JJ, Smith C, Crystal HA, Richardson J, Golub ET, et al (2011) Relationship of ethnicity, age, education, and reading level to speed and executive function among HIV+ and HIV- women: the Women’s Interagency HIV Study (WIHS) Neurocognitive Substudy. J Clin Exp Neuropsychol 33: 853–863. 21950512 10.1080/13803395.2010.547662 PMC3383771

[23] Falkenstein IA, Bartsch DU, Azen SP, Dustin L, Sadun AA, et al (2008) Multifocal electroretinography in HIV-positive patients without infectious retinitis. Am J Ophthalmol 146: 579–588. 18280451 10.1016/j.ajo.2007.12.021 PMC2614872

[24] Goldbaum MH, Falkenstein I, Kozak I, Hao J, Bartsch DU, et al (2008) Analysis with support vector machine shows HIV-positive subjects without infectious retinitis have mfERG deficiencies compared to normal eyes. Trans Am Ophthalmol Soc 106: 196–204. 19277235 PMC2646437

[25] Letendre S, Marquie-Beck J, Capparelli E, Best B, Clifford D, et al (2008) Validation of the CNS Penetration-Effectiveness rank for quantifying antiretroviral penetration into the central nervous system. Arch Neurol 65: 65–70. 18195140 10.1001/archneurol.2007.31 PMC2763187

[26] Kalyani PS, Holland GN, Fawzi AA, Arantes TE, Yu F, et al. (2012) Association between retinal nerve fiber layer thickness and abnormalities of vision in people with human immunodeficiency virus infection. Am J Ophthalmol 153: 734–742, 742 e1. 10.1016/j.ajo.2011.09.019 PMC4121665 22245459

[27] Tenhula WN, Xu SZ, Madigan MC, Heller K, Freeman WR, Sadun AA (1992) Morphometric comparisons of optic nerve axon loss in acquired immunodeficiency syndrome. Am J Ophthalmol 113: 14–20. 1370189 10.1016/s0002-9394(14)75746-0

[28] Gomez ML, Mojana F, Bartsch DU, Freeman WR (2009) Imaging of long-term retinal damage after resolved cotton wool spots. Ophthalmology 116: 2407–2414. 19815278 10.1016/j.ophtha.2009.05.012 PMC4172325

